# Dietary Lipid Sources Influence Fatty Acid Composition in Tissue of Large Yellow Croaker (*Larmichthys crocea*) by Regulating Triacylglycerol Synthesis and Catabolism at the Transcriptional Level

**DOI:** 10.1371/journal.pone.0169985

**Published:** 2017-01-12

**Authors:** Hong Qiu, Min Jin, Yi Li, You Lu, Yingmei Hou, Qicun Zhou

**Affiliations:** Laboratory of Fish Nutrition, School of Marine Sciences, Ningbo University, Ningbo, China; Universidade de Vigo, SPAIN

## Abstract

An 8-week feeding trial was conducted to evaluate the effects of dietary lipid sources on growth performance, fatty acid composition, rate-limiting enzyme activities and gene expression related to lipid metabolism in large yellow croaker (*Larmichthys crocea*). Five iso-nitrogenous and iso-lipidic experimental diets were formulated to contain different lipid sources, such as fish oil (FO), soybean oil (SO), linseed oil (LO), rapeseed oil (RO) and peanut oil (PO), respectively. Triplicate groups of 50 fish (initial weight 13.77±0.07g) were stocked in 15 floating net cages (1.5m×1.5m×2.0m). Fish fed the diets containing RO and LO had lower weight gain and specific growth rates than those fed the FO, SO and PO diets. Survival, feed efficiency, protein efficiency ratio, hepatosomatic index, viscerasomatic index and condition factor were not significantly affected by different dietary lipid sources. Fish fed the diet containing FO had higher lipid content in whole body compared with the other groups, whereas fish fed the SO diet had the lowest muscle lipid content. Fatty acid profiles of muscle and liver reflected the fatty acid composition of the diets. Plasma glucose, triglyceride, and the enzymatic activity of aspartate aminotransferase and alanine aminotransferase were significantly influenced by different dietary lipid sources, while total protein, cholesterol, superoxide dismutase or malondialdehyde in plasma were not affected by the different dietary lipid sources. Fish fed the LO diet had lower adipose triglyceride lipase and fatty acid synthase activities in liver than those fed the diets containing FO and RO, while the LO diet resulted in the highest hepatic carnitine palmitoultransferase-1 activity. Hepatic gene relative expression of adipose triglyceride lipase and carnitine palmitoyltransferase-1 in fish fed PO diet was significantly higher than all other groups, whereas fish fed the SO and LO diets had lower relative expression levels of lipoprotein lipase than the other groups. The highest relative expression levels of fatty acid synthase and acyl-CoA diacylglycerol acyltransferase-2 were observed in the FO group, while the highest relative expression of glucose 6-phosphate dehydrogenase occurred in fish fed the FO and RO diets. In summary, based on the growth performance, FO and SO appear to be suitable lipid sources for large yellow croaker, with the findings of this study also providing a molecular insight into the role of lipid metabolic mechanism in response to different dietary lipid sources.

## Introduction

Lipid serves two important roles in dietary formulations, as a high-density energy supply as well as being a source of essential fatty acids (EFA) [1]. Fish oil is the most important lipid source used in aquafeeds due to the presence of the long-chain polyunsaturated fatty acids (LC-PUFA), eicosapentanoic (EPA) and docosahexaenoic acid (DHA), which together can satisfy the EFA requirements of all fish species, especially marine fish species [2]. Over the past 30 years, around 20–25 million tonnes (Mt) of feed fish have been caught annually to produce about 6–7 Mt of fishmeal and 1.0–1.4 Mt of fish oil. In 2006, aquaculture consumed almost 89% of the available fish oil (FO) [3]. However, there is no realistic prospect of FO production being increased in the future with an increased competition for these small pelagic species for direct human consumption [1]. Therefore, the limiting supply of FO together with the rapid growth of aquaculture industry has resulted in increased feed costs, thus it is necessary to find suitable lipid sources to substitute FO use in aquafeeds.

Oils from terrestrial plants, specifically the oilseed crops producing vegetable oil (VOs), have almost no limitations to their supply, although their production and use raise some environmental and nutritional issues [1, 4]. Compared with FO, VOs are easy to obtain and store and are recognized as being the most potential lipid sources which can partly or totally replace FO in aquafeed [5]. The production of VOs exceeds 135 Mt with the four major oils: palm (42.4 Mt), soybean (37.7 Mt), rapeseed /canola (19.4 Mt), and sunflower (10.1 Mt), which accounted for more than 80% of the total global production [6]. In recent years, many studies have been performed to evaluate the effects of complete or partial replacement of dietary FO with VOs for freshwater and marine fish species [1]. While the partial replacement of FO with VOs did not affect growth and feed utilization [7–16], the total replacement of dietary FO with VOs significantly impacted upon growth and fillet quality resulting in growth retardation, abdominal and hepatic fat deposition [17]. However, for some fish species, it has been shown that fish oil can be totally replaced by VOs without affecting growth performance [18–22].

Several studies in fish have reported that dietary lipid sources could regulate gene expression and the activity of key enzymes involved in lipid metabolism, including anabolic and catabolic processes, as well as the relevant transcription factors [23]. Indeed, some studies demonstrated that replacing dietary FO by VOs could regulate the activity of enzymes and gene expression in anabolic process, such as glucose-6-phosphate dehydrogenase (*g6pd*) and fatty acid synthase (*fas*) [5, 24–27]. Furthermore, the enzymatic activities and gene expression related to catabolic processes could also be affected by dietary VOs, such as carnitine palmitoyltransferase-1 (*cpt-1*) and lipoprotein lipase (*lpl*) [5, 28–29]. Moreover, acyl-CoA diacylglycerol acyltransferase (DGAT) and adipose triglyceride lipase (ATGL) are known to be key enzymes involved in the mechanisms of intracellular enzyme synthesis and degradation of triacylglycerol, respectively [30–32].

Large yellow croaker (*Larmichthys crocea*) is mainly distributed in the northern South China sea, East China sea and Taiwan strait. It is widely cultured in south-eastern China coastal areas, especially in Fujian and Zhejiang Provinces [33]. Studies on the lipid nutrition of this fish have been conducted intensively in the past few years [4, 34–37]. Yi et al. [38] reported that there was no significant effect of dietary FO replacement by rapeseed oil on growth and body composition, although the fatty acid compositions of muscle and skin color were significantly influenced by dietary lipid sources. To our knowledge, no information is available on the mechanisms regarding dietary lipid sources regulating lipid transport, synthesis and catabolism for this fish species yet. Therefore, the objectives of this study are to evaluate the effects of different dietary lipid sources on growth performance, hepatic gene expression and rate-limiting enzymatic activities related to lipid metabolism, and to explore the metabolic mechanisms by which dietary lipid sources regulate lipid deposition and transportation at the transcriptional level. The results may be helpful in developing resource-saving and environmentally friendly artificial feeds for large yellow croaker.

## Materials and Methods

### Ethics statement

The present study was performed in strict accordance with the Standard Operation Procedures (SOPs) of the Guide for Use of Experimental Animals of Ningbo University. All animal care and use procedures were approved by the Institutional Animal Care and Use Committee of Ningbo University. Fish were anesthetized with tricaine methane sulfonate (MS-222) to minimize suffering before being assigned to cages and sampling.

### Diet and feeding trial

Peruvian fishmeal, soybean meal, soybean protein concentrate, squid meal and wheat gluten meal were used as protein sources, starch was used as carbohydrate source. Five iso-nitrogenous (crude protein about 45.0%) and iso-lipidic (about 10.0%) experimental diets were formulated to contain fish oil (FO), soybean oil (SO), linseed oil (LO), rapeseed oil (RO) and peanut oil (PO), respectively (Table 1). Each lipid source was supplemented at 3.40% in the diet. Feed procedure was performed as described in our previous study [33]. Formulation, proximate composition of the experimental diets and fatty acid composition of lipid sources are presented in Tables 1–3, respectively.

**Table 1:** Formulation and proximate composition of the experimental diets.

Ingredient (g/100g)	Dietary lipid sources				
	FO	SO	LO	RO	PO
Peru fish meal^1^	41.0	41.0	41.0	41.0	41.0
Soybean meal^1^	5.0	5.0	5.0	5.0	5.0
Soybean protein concentrate^1^	7.3	7.3	7.3	7.3	7.3
Wheat gluten meal^1^	10.0	10.0	10.0	10.0	10.0
Squid meal^1^	2.0	2.0	2.0	2.0	2.0
α-Starch^1^	25.0	25.0	25.0	25.0	25.0
Soybean lecithin^1^	2.0	2.0	2.0	2.0	2.0
Fish oil^1^(FO)	3.4				
Soybean oil^2^(SO)		3.4			
Linseed oil^3^(LO)			3.4		
Rapeseed oil^4^(RO)				3.4	
Peanut oil^4^(PO)					3.4
Vitamin premix^5^	1.5	1.5	1.5	1.5	1.5
Mineral premix^5^	1.5	1.5	1.5	1.5	1.5
Ca(H_2_PO_4_)_2_^1^	1.0	1.0	1.0	1.0	1.0
Choline chloride^1^	0.3	0.3	0.3	0.3	0.3
Proximate composition (g/100g)					
Crude protein	45.1	45.1	45.2	45.5	45.1
Crude lipid	10.3	10.3	10.3	10.2	10.2
Moisture	7.8	8.1	8.1	8.5	8.3
Ash	10.1	10.1	10.4	9.8	10.0

^1^Peru fish meal, soybean meal, soybean protein concentrate, wheat gluten meal, squid meal, α-starch and soybean lecithin were supplied by Ningbo Tech-bank Aqua feed company, Ningbo, China.

^2^Soybean oil (SO) was bought from Kerry grain and oil Co., Ltd. Shanghai, China.

^3^Linseed oil (LO) was bought from Changbaigongfang Co., Ltd. Jilin, China.

^4^Rapeseed oil (RO) and peanut oil (PO) were bought from Luhua Co., Ltd. Shanghai, China

^5^Vitamin premix and mineral premix were described as Zhou et al. [33]

**Table 2:** Fatty acid composition of lipid sources (% total fatty acid).

Fatty acids	FO	SO	LO	RO	PO
14:0	5.77	0.10	-	-	0.10
15:0	0.72	-	-	-	-
16:0	16.16	10.99	5.67	3.24	11.66
17:0	0.51	-	-	-	-
18:0	2.57	4.07	4.33	1.5	1.90
20:0	-	0.28	-	0.59	1.74
22:0	-	0.38	-	-	1.16
24:0	-	-	-	-	1.49
ΣSFAs	25.73	15.82	10.00	5.33	18.05
16:1n-7	8.08	0.20	0.20	-	0.10
18:1n-6	-	-	-	-	-
18:1n-7	-	-	-	-	-
18:1n-9	20.56	23.63	22.27	60.76	44.26
20:1n-9	7.88	0.1	-	1.23	0.84
22:1n-9	11.09	-	-	0.27	-
24:1n-9	0.51	-	-	-	-
ΣMUFAs	48.12	23.93	22.57	62.26	45.20
18:2n-3	-	-	-	-	0.12
18:3n-3	1.45	5.90	49.51	10.16	-
20:3n-3	-	-	-	-	0.06
20:5n-3(EPA)	8.82	-	-	-	-
22:6n-3(DHA)	7.85	-	-	-	-
Σn-3 PUFAs	18.12	5.90	49.51	10.16	0.18
18:2n-6	0.47	51.76	12.89	19.04	32.4
20:2n-6	0.29	-	-	-	-
22:4n-6	0.79	-	-	-	-
Σn-6 PUFAs	1.55	51.76	12.89	19.04	32.4
n-3/n-6 PUFA	11.69	0.11	3.84	0.53	0.01

FO: fish oil; SO: soybean oil; LO: linseed oil; RO: rapeseed oil; PO: peanut oil

SFAs: saturated fatty acids;

MUFAs: mono-unsaturated fatty acids;

PUFAs: poly-unsaturated fatty acids;

“-”: non-detected

**Table 3:** Fatty acid composition of the experimental diets (% total fatty acid).

Fattyacids	FO	SO	LO	RO	PO
14:0	4.21	2.74	2.60	2.59	2.57
16:0	16.39	15.01	13.32	12.87	14.63
18:0	4.39	4.61	4.39	3.64	4.45
20:0	0.49	0.36	0.28	0.50	0.97
22:0	nd	0.34	nd	0.33	1.52
24:0	nd	0.28	0.28	0.18	0.80
ΣSFAs	26.86	24.17	21.52	20.75	25.58
16:1n-7	4.69	2.60	2.44	2.44	2.42
18:1n-9	17.21	18.94	18.07	30.06	24.17
20:1n-7	5.51	4.35	4.44	4.87	4.64
22:1n-9	8.79	6.26	6.52	6.56	6.35
ΣMUFAs	37.38	32.77	32.19	44.61	38.28
18:3n-3	1.98	3.55	16.88	3.99	1.52
18:4n-3	1.61	0.94	1.00	0.95	0.93
20:4n-3	0.72	0.33	0.34	0.34	0.33
20:5n-3(EPA)	6.01	3.47	3.49	3.45	3.45
22:5n-3	0.76	0.51	0.78	0.74	0.81
22:6n-3(DHA)	10.78	8.07	7.71	7.95	7.86
Σn-3 PUFAs	21.86	16.87	30.20	17.42	14.90
18:2n-6	12.48	25.89	15.76	16.90	20.91
20:4n-6	0.81	0.33	0.34	0.33	0.33
Σn-6 PUFAs	13.91	26.22	16.10	17.23	21.24
n-3/n-6 PUFA	1.57	0.64	1.88	1.01	0.70
DHA/EPA	1.79	2.33	2.21	2.30	2.27

FO: fish oil; SO: soybean oil; LO: linseed oil; RO: rapeseed oil; PO: peanut oil

SFAs: saturatedfattyacids;

MUFAs: mono-unsaturatedfattyacids;

PUFAs: poly-unsaturatedfattyacids;

nd: non-detected

Large yellow croaker were obtained from a local hatchery farm (Ning-Gang Aquatic Fingerlings Limited Company of Xiangshan Bay, Ningbo, China) located at [121.752E, 29.545N]. The fish were reared and fed a commercial diet(45.0% crude protein and 10.0% crude lipid, Ningbo Tech-bank Aquafeed company, Ningbo, China) for two weeks to acclimate to the experimental conditions. Before the feeding trial, fish were fasted for 24 h and weighed. Fish (initial weight was13.77±0.07g) were randomly assigned to 15 outdoor net cages (1.5m×1.5m×2.0m) with 50 individuals in each cage. Each diet was randomly distributed in triplicate cages. Fish were hand-fed twice daily to apparent satiation (05:00 and17:00), and the amount of diet consumption in each net cage was recorded daily. The feeding trial lasted for 8 weeks. During the experimental period, water temperature ranged from 26.5 to 31.5℃, salinity ranged from 19 to 25‰, and dissolved oxygen was not less than 7.0mg/L.

### Samples collection

At the termination of the trial, fish in each net cage were anesthetized with MS-222 (Shanghai Reagent Corp., Shanghai, China), and then individually weighed, counted and sampled. Hepatosomatic index (HSI), viscerosomatic index (VSI) and condition factor (CF) were determined from four individual fish per net cage by obtaining tissues (viscera and liver) and expressing ratios as a percent of body weight, before removing muscle to analyze for proximate composition. Three fish from each cage were randomly sampled to analyze the proximate composition of whole body. Blood was sampled from the caudal vein of six fish from each net cage using ethylenediaminetetraacetic acid (EDTA) containing Vacutainers (HuaboMedical Instrument Co., Ltd, Heze, China). Plasma was separated from the blood *via* centrifugation (at 956 g, 4°C, 10 min) and stored at -80°C until use. The liver from eight fish in each cage were pooled into two 1.5 ml tubes randomly, frozen in liquid N_2_ and then stored at -80°C for analysis of gene expression related to lipid metabolism. Liver from another three fish per net cage were sampled, frozen in liquid N_2_ and then stored at -80°C for analysis of the enzymatic activity. Liver and muscle were further sampled from three fish per net cage to analyze the fatty acid composition.

### Chemical analysis

Crude protein, crude lipid, dry matter and ash content in diets, whole body and muscle of the fish were determined according to the methods of the Association of Official Analytical Chemists [39]. Dry matter content was determined by drying the samples to a constant weight at 105°C. The crude protein (N × 6.25) was determined using the Dumas combustion methods with a protein analyzer (FP-528, Leco, USA). Crude lipid was determined by the ether extraction method using Soxtec System HT (Soxtec System HT6, Tecator, Sweden) and ash content was determined using a muffle furnace at 550°C for 8 h.

### Fatty acid determination

The fatty acid profile of diets and fish tissues (liver and muscle) were determined as described by [34] with few modifications. The freeze-dried samples (~80mg liver sample and ~120mg muscle sample) were added into a 12 ml volumetric glass screwed tube with lid (contain a teflon gasket). Then 3 ml potassium hydroxide methanol (1 N) was added and heated in a water bath at 72°C for 20 min. After cooling, 3 ml HCL–methanol (2 N) was added and the mixture was heated at 72°C in a water bath for another 20 min. Previous tests were conducted to make sure that all fatty acids can be esterified following the procedures above. Finally, 1 ml hexane was added to the mixture above, shaken vigorously for 1 min, and then allowed to separate into two layers. Fatty acid methyl esters were separated, and measured by GC-MS (Agilent technologies 7890B -5977A, USA). Results are presented as a percentage of total fatty acids.

### Plasma biochemical analysis

Plasma samples from each cage were pooled. Plasma characteristics were determined according to the method described previously [33]. Glucose, total protein, triglyceride, cholesterol concentrations in plasma were determined by automatic chemistry analyzer (Hitachi 7600–110, Tokyo, Japan). The activities of aspartate aminotransferase (AST), alanine aminotransferase (ALT) and superoxide dismutase in plasma were measured using the diagnostic reagent kit purchased from the Nanjing Jiancheng Bioengineering Institute (Nanjing, China), according to the manufacturer`s instructions. Lipid peroxidation, such as malondialdehyde was described previously [33].

### Hepatic lipid metabolism enzymes

ATGL, CPT-1 and FAS are key enzymes for lipid metabolism in fish. Livers were thawed and homogenized in nine volumes of ice-cold buffer (10 mM HEPES, 1 mM EDTA, 1 mM dithiothreitol, pH 7.4). The extract was later centrifuged at 850×g at 4°C for 10 min. The supernatant was used to determine the activities using Elisa lipase assay kit for fish from Yuan Ye Biological Company (Shanghai, China). Hepatic ATGL, CPT-1 and FAS activities were analyzed according to the manufacturer's instructions.

### RNA extraction and real-time quantitative PCR

Total RNA was extracted from the liver of juvenile large yellow croaker using TRIzol reagent (Transgen, Beijing, Chin) according to the manufacturer’s instructions. Isolated RNA quantity and quality were determined via spectrophotometry using a Nano Drop 2000 spectrophotometer and on a 1.2% denaturing agarose gel, respectively. The cDNA was generated from 1000 ng of DNAase treated RNA and synthesized by a PrimeScript^™^ RT Reagent Kit with gDNA Eraser (perfect realtime) (Transgen, Beijing, China) according to the manufacturer’s instructions. *β-actin* was used as the house-keeping gene and the stability of *β-actin* expression was confirmed. Specific primers of *lpl*, *cpt-1*, *atgl*, *dgat-2*, *fas* and *g6pd* used for RT-qPCR were designed using Primer Premier 5.0 software (Table 4). Amplification was performed using a quantitative thermal cycler (Roche, Lightcycler 96, Switzerland). PCR measurements were performed in a total volume of 20μL, containing 1.0μL of each primer, 10μL of 2×conc SYBR Green I Master (Roche, Switzerland), 2μL of cDNA and 6μL DEPC-water. The procedure for quantitative PCR was employed: 95°C for 2 min, followed by 45 cycles of 95°C for 10s, 58°C for 10 s and 72°C for 20s. Standard curves were generated using six different dilutions (in triplicate) of the cDNA samples, and the amplification efficiency was analyzed as follows: E = 10^(–1/Slope)^-1. The amplification efficiencies of all genes were approximately equal and ranged from 93 to 102%. Meanwhile, the expression levels of the target genes were calculated using the 2^–ΔΔCt^ method described by Livak and Schmittgen [40]. At the end of each PCR amplification, melting curve analysis was performed to confirm that only one PCR product was present. Normalized gene expression for the control diet group (FO diet) was set at 1.

**Table 4:** Primers pair sequences for real-time PCR.

Gene	Forward (5’-3’)	Reverse (5’-3’)	Reference
*fas*	ACTCCTATGTGGCAGCATAGAC	GTTTCAGCCTCAGACTCTTTGCC	JX456351
*g6pd*	TATCTTTGCCGAACGCTGTC	TGTCTCCTCTGGGCTGAAGT	XM010731711
*atgl*	CCATGCATCCGTCCTTCAACC	GAGATCCCTAACCGCCCACT	Yan et al. [37]
*cpt-1*	GCTGAGCCTGGTGAAGATGTTC	TCCATTTGGTTGAATTGTTTACTGTCC	Yan et al. [37]
*lpl*	GCGGAAACACAGACCTTCAT	AGTCGGCACACGCTCATAG	JQ327827
*dgat-2*	TTCGGTGCTTTCTGCAACTTCG	AAGGATGGGGAAGCGGAAGT	Yan et al. [37]
*β-actin*	CTACGAGGGTTAGCCCTGCC	TGAAGGAGTAACCGCGCTCTGT	GU584189

*lpl*: lipoprotein lipase;

*atgl*: adipose triglyceride lipase;

*cpt-1*: carnitine palmitoyltransferase-1;

*dgat-2*: acyl CoA diacylglycerol acyltransferase 2;

*fas*: fatty acid synthase;

*g6pd*: glucose 6-phosphate dehydrogenase

### Statistical analysis

Results are presented as mean and standard deviation (S.D). Data were analyzed *via* one-way analysis of variance (ANOVA) followed by Tukey’s multiple-range test. When the homogeneity of variance was not satisfied, an independent-samples t-test was performed to compare the differences. The level of significance was set at *P*<0.05. All statistical analysis was performed using SPSS 16.0 for Windows (SPSS Inc., Michigan Avenue, Chicago, IL, USA).

## Results

### Growth performance, feed utilization and morphologic index

The effects of different dietary lipid sources on growth performance, survival, feed utilization and morphologic index are presented in Table 5. Survival ranged from 94.67% to 98.00%, with no significant difference in survival rate among treatments. Fish fed the FO, SO and PO diets had a significantly higher weight gain (WG) and specific growth rate (SGR) than those fed the diets containing LO and RO (*P*<0.05). However, feed intake, feed efficiency (FE) and protein efficient rate (PER) were not influenced by the dietary lipid sources (*P*>0.05). There was no significant difference in HSI, VSI or CF among different lipid sources (*P*>0.05), although fish fed the LO diet displayed the lowest CF, HSI and VSI among all the treatments.

**Table 5:** Growth performance, feed utilization and morphologic index of large yellow croaker fed diets containing different lipid sources.

Dietary lipid sources	Initial weight (g)	WG (%)^1^	SGR(%day^-1^)^2^	Survival (%)^3^	Feed intake^4^	FE^5^	PER^6^	HSI (%)^7^	VSI(%)^8^	CF(gcm^-3^)^9^
**FO**	13.78±0.01	189.95±5.83^b^	1.97±0.04 ^b^	96.00±4.00	0.62±0.02	0.58±0.07	1.31±0.16	1.55±0.23	3.03±0.20	1.84±0.05
**SO**	13.78±0.04	193.97±3.87^b^	2.00±0.03^b^	94.67±6.11	0.63±0.01	0.59±0.08	1.39±0.10	1.75±0.24	3.27±0.50	1.71±0.06
**LO**	13.77±0.02	181.60±6.86^a^	1.92±0.04^a^	98.00±3.46	0.61±0.02	0.55±0.04	1.29±0.04	1.41±0.10	2.78±0.42	1.69±0.07
**RO**	13.82±0.07	181.46±3.70^a^	1.92±0.02 ^a^	97.33±4.62	0.60±0.081	0.52±0.10	1.23±0.10	1.64±0.08	3.57±0.04	1.72±0.14
**PO**	13.78±0.04	192.91±4.89^b^	1.99±0.03^b^	98.00±3.46	0.64±0.05	0.62±0.04	1.38±0.08	1.57±0.16	3.01±0.31	1.85±0.09
**ANOVA *P-value***	0.689	0.031	0.036	0.86	0.75	0.517	0.377	0.244	0.129	0.11

Values are represented as the means of three replicates. Means in the same column with different superscripts are significantly different (P < 0.05).

FO: fish oil; SO: soybean oil; LO: linseed oil; RO: rapeseed oil; PO: peanut oil

^1^Weight gain (WG, %) = 100× (W_t_− W_i_)/ W_i_

^2^Specific growth ratio (SGR, % day^-1^) = 100 × (Ln W_t_−Ln W_i_) / t

^3^Survival (%) = 100 ×(final number of fish) / (initial number of fish)

^4^Feed intake (g day^-1^) = feed consumption (g)/(days × (final body weight + initial body weight)/2)

^5^Feed efficiency (FE) = weight gain (g, wet weight) / feed consumed (g, dry weight)

^6^Protein efficiency ratio (PER) = weight gain (g, wet weight) / protein intake (g, dry weight)

^7^Hepatosomatic index (HSI,%) = 100 × liver wet weight (g)/body wet weight (g)

^8^Viscerosomatic index (VSI,%) = 100 × viscerosomatic weight (g) / body wet weight (g)

^9^Condition factor (CF,g·cm^-3^) = 100 ×body wet weight (g) / body length (cm)^3^

### Proximate composition in whole body and muscle

Effects of different dietary lipid sources on proximate composition of the whole body and muscle are presented in Table 6. Dry matter and ash contents in whole body were not significantly affected by the dietary lipid sources (*P*>0.05). Fish fed the FO diet had a significantly higher whole body lipid content than those fed the other diets (*P*<0.05), with the lowest whole body lipid content occurred in fish fed the LO and RO diets. Fish fed the diet containing LO had the highest whole body protein content among all the treatments. Fish fed the diet containing SO had lower crude lipid content in muscle than those fed the other diets. Moreover, dry matter, crude protein and ash contents in muscle were not significantly influenced by the dietary lipid sources (*P*>0.05).

**Table 6:** Proximate composition in whole body and muscle of the large yellow croaker fed diets containing different lipid sources.

Index	FO	SO	LO	RO	PO	ANOVA *P-value*
**Whole body (%)**						
**Dry matter**	29.02±2.30	27.96±0.84	26.86±0.45	26.6±0.92	28.57±0.54	0.134
**Crude protein**	14.86±0.08^a^	15.43±0.35^ab^	15.72±0.11^b^	15.36±0.32^ab^	14.86±0.10^a^	0.003
**Crude lipid**	11.96±0.53^c^	9.57±0.71^b^	7.82±0.63^a^	7.16±0.08^a^	10.19±0.42^b^	0.000
**Ash**	11.65±1.19	11.91±1.51	12.43±0.55	13.13±0.65	13.32±2.32	0.542
**Muscle (%)**						
**Dry matter**	28.67±0.31	27.16±2.22	28.74±0.12	28.05±1.02	29.09±1.26	0.548
**Crude protein**	17.17±0.43	16.91±0.63	17.37±0.08	17.12±0.10	17.18±0.54	0.243
**Crude lipid**	9.9±0.55^b^	7.92±0.03^a^	9.90±0.32^b^	9.15±0.7^b^	10.12±0.61^b^	0.002
**Ash**	4.22±0.08	4.31±0.23	4.16±0.07	4.42±0.18	4.36±0.14	0.285

Values are represented as the means of three replicates. Means in the same row with different superscripts are significantly different (*P*< 0.05).

FO: fish oil; SO: soybean oil; LO: linseed oil; RO: rapeseed oil; PO: peanut oil

### Fatty acid composition of liver and muscle

Effects of the different dietary lipid sources on fatty acid composition in liver and muscle are shown in Tables 7 and 8 respectively. The fatty acid profiles of the liver and muscle reflected that of the diet. Fish fed the diets containing SO, RO and PO had a significantly lower total n-3 PUFA content than those fed the FO and LO diets (*P*<0.05). The highest 18:3n-3 content in liver was observed in fish fed the LO diet, and the highest hepatic EPA and DHA contents were found in fish fed the FO diet. Fish fed the FO diet had a significantly lower total n-6 PUFA liver content than those fed the other diets. However, there was no significant difference in total saturated fatty acids (SFA) or DHA/EPA ratio in the liver among all treatments (*P*>0.05).

**Table 7:** Hepatic fatty acid composition of the large yellow croaker fed diets containing different lipid sources (%, of total fatty acid).

Fatty acids	FO	SO	LO	RO	PO	ANOVA *P-value*
14:0	2.48±0.01^c^	2.16±0.12^b^	1.94±0.06^a^	2.13±0.05^ab^	2.06±0.09^ab^	0.000
16:0	21.16±0.38	18.77±0.71	18.89±1.27	19.47±1.1	18.83±1.02	0.054
18:0	10.15±0.99	10.88±0.77	10.16±0.73	12.5±0.64	11.08±1.22	0.050
20:0	0.33±0.03^ab^	0.30±0.01^ab^	0.17±0.15^a^	0.41±0.03^b^	0.49±0.03^b^	0.003
∑SFA	35.21±1.06	32.90±1.16	31.97±1.43	35.19±1.30	33.73±1.91	0.068
16:1n-7	11.17±0.37^b^	9.56±1.05^ab^	9.48±0.45^ab^	9.56±1.02^ab^	9.09±0.46^a^	0.045
18:1n-9	26.42±0.31^a^	27.02±1.06^a^	25.72±0.34^a^	31.04±0.59^c^	28.86±0.67^b^	0.000
20:1n-7	3.9±0.21	3.56±0.40	3.45±0.26	3.37±0.28	3.77±0.32	0.303
22:1n-9	2.9±0.35	2.76±0.33	2.69±0.19	2.56±0.22	2.67±0.42	0.699
∑MUFA	45.68±2.32^ab^	43.79±1.45^ab^	42.19±0.31^a^	47.27±1.09^b^	45.21±0.60^ab^	0.013
18:3n-3	1.09±0.13^a^	1.93±0.26^a^	8.76±0.99^b^	1.47±0.29^a^	1.18±0.27^a^	0.000
18:4n-3	0.56±0.04^b^	0.33±0.01^a^	0.41±0.07^a^	0.36±0.06^a^	0.32±0.04^a^	0.000
20:4n-3	0.46±0.02^c^	0.30±0.01^b^	0.25±0.22^a^	0.31±0.02^a^	0.31±0.01^a^	0.000
20:5n-3 (EPA)	2.41±0.07^b^	1.32±0.01^a^	1.58±0.05^a^	1.34±0.17^a^	1.55±0.12^a^	0.000
22:5n-3	1.01±0.13^b^	0.40±0.13^a^	0.51±0.21^ab^	0.46±0.39^ab^	0.52±0.28^ab^	0.039
22:6n-3 (DHA)	5.29±0.27^b^	3.21±0.15^a^	3.66±0.53^a^	3.54±0.49^a^	3.93±0.34^a^	0.001
∑n-3 PUFA	10.82±0.43^b^	7.49±0.38^a^	15.17±0.96^c^	7.48±1.43^a^	7.81±0.79^a^	0.000
18:2n-6	7.41±0.32^a^	14.81±1.53^c^	9.82±0.82^ab^	9.64±0.87^ab^	12.19±1.7^bc^	0.000
20:2n-6	0.31±0.01^a^	0.46±0.06^b^	0.33±0.04^a^	0.27±0.06^a^	0.36±0.01^ab^	0.004
20:4n-6	0.60±0.03^a^	0.34±0.01^b^	0.37±0.02^b^	0.35±0.05^b^	0.37±0.07^b^	0.000
∑n-6 PUFA	8.32±0.33^a^	15.85±1.59^c^	10.52±0.83^ab^	10.26±0.97^ab^	12.92±1.71^bc^	0.000
n-3/n-6 PUFA	1.30±0.06^c^	0.48±0.02^a^	1.44±0.03^d^	0.69±0.08^b^	0.61±0.03^b^	0.000
DHA/EPA	2.20±0.07	2.43±0.10	2.32±0.40	2.63±0.05	2.54±0.02	0.111

Values are represented as the means of three replicates. Means in the same row with different superscripts are significantly different (P < 0.05).

FO: fish oil; SO: soybean oil; LO: linseed oil; RO: rapeseed oil; PO: peanut oil

**Table 8:** Fatty acid composition in muscle of the large yellow croakerfed diets containing different lipid sources (%, of total fatty acid).

Fatty acids	FO	SO	LO	RO	PO	ANOVA *P-value*
14:0	3.24±0.07^a^	2.45±0.11b	2.45±0.03^b^	2.46±0.11^b^	2.56±0.05^b^	0.000
16:0	14.91±0.51^a^	14.08±0.53^ab^	13.26±0.29^b^	13.53±0.36^b^	14.03±0.76^ab^	0.024
18:0	6.88±0.33	7.73±0.71	6.54±0.39	7.01±0.65	7.47±0.68	0.159
20:0	1.26±0.60	1.08±0.49	1.04±0.5	1.44±0.05	1.70±0.13	0.338
∑SFA	27.87±0.38^c^	26.82±0.32^bc^	24.73±0.49^a^	25.65±0.69^ab^	27.92±0.34^c^	0.000
16:1n-7	6.49±0.06^b^	5.90±0.39^ab^	5.13±0.64^a^	5.48±0.45^ab^	6.07±0.2^ab^	0.016
18:1n-9	19.66±0.62^a^	21.36±1.28^ab^	19.33±0.67^a^	26.43±0.88^c^	22.96±0.94^b^	0.000
20:1n-7	4.51±0.44	3.84±0.45	3.8±0.33	4.19±0.17	3.93±0.23	0.135
22:1n-9	5.97±0.48	5.08±0.11	5.31±0.22	5.11±0.45	5.36±0.31	0.057
∑MUFA	38.00±1.16^b^	37.14±1.79^ab^	34.50±1.43^a^	42.15±0.27^c^	39.31±0.57^bc^	0.000
18:3n-3	1.89±0.06^a^	2.93±0.10^b^	11.00±0.52^c^	3.30±0.19^b^	1.72±0.09^a^	0.000
18:4n-3	1.27±0.02^a^	0.82±0.15^b^	0.82±0.04^b^	0.79±0.04^b^	0.87±0.14^b^	0.000
20:4n-3	0.98±0.08^b^	0.69±0.13^a^	0.77±0.02^ab^	0.67±0.15^a^	0.79±0.06^ab^	0.031
20:5n-3 (EPA)	4.97±0.17^a^	3.52±0.62^b^	3.46±0.07^b^	3.55±0.16^b^	3.56±0.27^b^	0.001
22:5n-3	1.75±0.10	1.32±0.28	1.48±0.19	1.46±0.08	1.43±0.08	0.078
22:6n-3 (DHA)	10.62±0.85^a^	8.36±1.32^b^	8.78±0.89^b^	8.49±0.47^b^	8.51±0.39^b^	0.043
∑n-3 PUFA	21.48±0.90^b^	17.64±1.95^a^	26.31±1.19^c^	18.26±0.70^a^	16.88±0.96^a^	0.000
18:2n-6	10.17±0.08^a^	16.11±0.37^c^	12.15±0.75^b^	12.42±0.65^b^	13.51±0.75^b^	0.000
18:3n-6	0.30±0.01^a^	0.22±0.00^b^	0.21±0.01^b^	nd	0.23±0.01^b^	0.000
20:4n-6	0.75±0.10^b^	0.53±0.08^a^	0.61±0.08^ab^	0.59±0.07^ab^	0.55±0.02^a^	0.031
∑n-6 PUFA	12.51±0.13^a^	18.05±0.11^c^	14.11±0.83^b^	13.87±0.98^b^	15.45±0.19^b^	0.000
n-3/n-6 PUFA	1.73±0.07^c^	0.99±0.12^a^	1.87±0.13^c^	1.27±0.14^b^	1.12±0.07^ab^	0.000
DHA/EPA	2.13±0.10	2.38±0.08	2.53±0.23	2.39±0.21	2.40±0.10	0.095

Values are represented as the means of three replicates. Means in the same row with different superscripts are significantly different (P < 0.05)

FO: fish oil; SO: soybean oil; LO: linseed oil; RO: rapeseed oil; PO: peanut oil.

The contents of SFA, MUFA, n-6 PUFA, n-3 PUFA and n-3/n-6 ratio in the muscle were significantly influenced by the dietary lipid sources(*P*<0.05). Fish fed the LO diet had a lower total MUFA content in muscle than those fed the other diets. The highest total n-3 PUFA content in muscle was observed in fish fed the LO diet, whereas fish fed diets containing SO, RO and PO had lower total n-3 PUFA muscle contents than those fed the FO and LO diets. However, fish fed the SO diet had a significantly higher total n-6 PUFA content than those fed the other diets (*P*<0.05).

### Hematology, hematological and hepatic lipid metabolic enzyme activities

The hematology, hematological and hepatic lipid metabolic enzyme activities of large yellow croaker fed different lipid sources are presented in Table 9. Fish fed the FO diet had significantly lower plasma triglyceride levels than those fed the LO, RO and PO diets (*P*<0.05). The highest plasma glucose concentration occurred in fish fed the LO diet. The highest plasma AST activity was observed in the RO group, with the lowest AST activity occurring in fish fed the FO diet. Fish fed the PO diet had a higher plasma ALT activity compared to all other groups.

**Table 9:** Hematological characteristics, enzyme activities in plasma and liver of large yellow croaker fed diets containing different lipid sources.

Item	FO	SO	LO	RO	PO	ANOVA *P-value*
**Hematological characteristics**						
**TP (g/L)**	26.85±0.35	26.27±0.67	26.97±0.32	26.90±0.10	26.60±0.10	0.415
**CHOL (mmol/L)**	3.05±0.86	3.01±0.59	3.68±0.44	3.13±0.52	3.21±0.34	0.641
**TG (mmol/L)**	3.32±0.82^a^	3.82±0.13^ab^	6.10±0.61^cd^	5.03±0.16^bc^	6.59±0.44^d^	0.000
**GLU (mmol/L)**	7.73±0.76^a^	6.97±0.05^a^	9.87±0.56^b^	8.85±1.62^b^	7.46±0.99^a^	0.023
**Enzyme activities in plasma**						
**SOD (U/ml)**	88.38±0.74	86.38±1.25	86.53±1.52	85.36±2.57	85.36±2.27	0.292
**MDA (nmol/ml)**	2.43±0.99	2.99±0.79	2.96±0.27	1.87±0.27	2.75±0.85	0.326
**AST (U/gprot)**	5.71±1.20^a^	9.66±0.35^b^	14.97±0.33^c^	16.77±0.53^d^	14.92±0.19^c^	0.000
**ALT (U/gprot)**	8.08±0.95^a^	6.63±1.01^a^	8.80±0.40^ab^	8.66±0.86^ab^	10.47±1.01^b^	0.005
**Hepatic lipid metabolic key enzyme activities**						
**ATGL (ng/g prot)**	250.78±26.56^bc^	200.00±25.78^ab^	152.34±9.38^a^	272.66±6.25^c^	265.89±28.99^c^	0.000
**CPT-1(U/g prot)**	369.85±8.44^ab^	362.27±8.02^a^	412.78±7.16^c^	358.40±11.66^a^	388.54±12.25^bc^	0.000
**FAS (U/g prot)**	4564.91±132.45^b^	4538.60±244.52^b^	3822.22±153.28^a^	4468.42±78.95^b^	4240.35±144.94^ab^	0.001

Values are represented as the means of three replicates. Means in the same row with different superscripts are significantly different (P < 0.05).

FO: fish oil; SO: soybean oil; LO: linseed oil; RO: rapeseed oil; PO: peanut oil.

TP: total protein; CHOL: cholesterol; TG: triglyceride; GLU: glucose; SOD: superoxide dismutase; AST: aspartate aminotransferase; ALT: alanine aminotransferase; MDA: methane dicarboxylic aldehyde; ATGL: Adipose triglyceride lipase; CPT-1: Carnitine palmitoyltransferase-1; FAS: fatty acid synthase.

Hepatic ATGL, CPT-1 and FAS activities were significantly affected by the different dietary lipid sources (*P*<0.05). The lowest ATGL and FAS activity were both observed in fish fed the LO diet. However, fish fed the LO diet had a significantly higher CPT-1 activity than those fed diets containing FO, SO and RO (*P*<0.05).

### Expression of gene involved in triacylglycerol synthesis and catabolism

**Fig 1:**
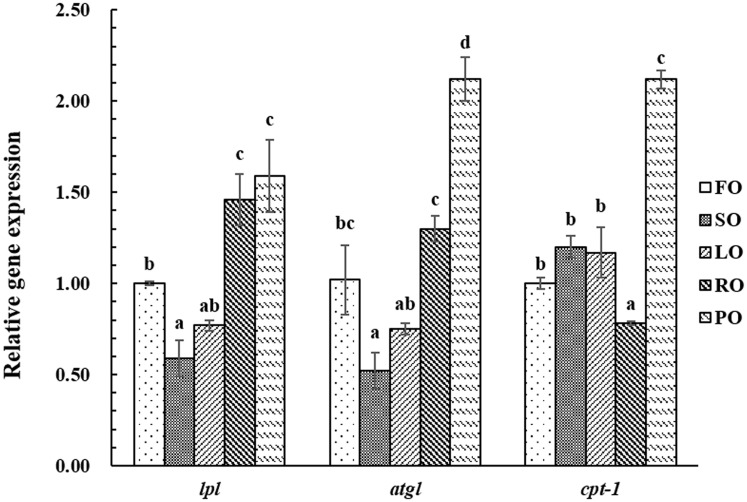
Effect of dietary lipid sources on relative expression of hepatic fatty acid oxidation-related genes in large yellow croaker. Values are means, bars bearing the same letters are not significantly different among treatments by Tukey’s test (*P*>0.05). *lpl*: lipoprotein lipase; *atgl*: adipose triglyceride lipase; *cpt-1*:carnitine palmitoyltransferase-1 FO: fish oil; SO: soybean oil; LO: linseed oil; RO: rapeseed oil; PO: peanut oil

**Fig 2:**
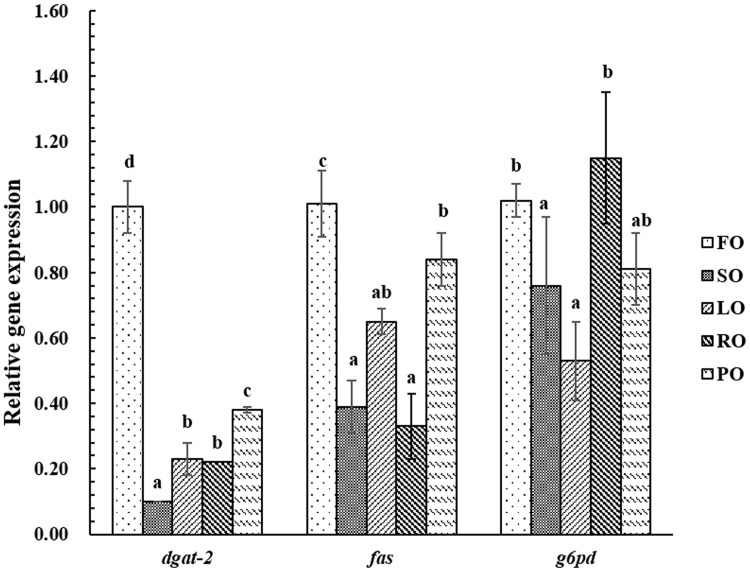
Effect of dietary lipid sources on relative expression of hepatic fatty acid synthetic-related genes in large yellow croaker. Values are means, bars bearing the same letters are not significantly different among all treatments by Tukey’s test (*P*>0.05). *dgat-2*: acyl CoA diacylglycerol acyltransferase 2; *fas*: fatty acid synthase; *g6pd*: glucose 6-phosphate dehydrogenase FO: fish oil; SO: soybean oil; LO: linseed oil; RO: rapeseed oil; PO: peanut oil

Relative expression of genes involved in triacylglycerol synthesis and catabolism in liver of large yellow croaker fed different dietary lipid sources are shown in Figs 1 and 2. The lowest relative expression of *lpl* was recorded in fish fed the SO diet, whereas the highest relative expression of *lpl* was observed in fish fed the diets containing PO and RO. Fish fed the diet containing PO had significantly higher hepatic expression of *atgl* and *cpt-1* than those fed the other diets (*P*<0.05). Fish fed the diets containing SO and LO had lower relative expression of hepatic *g6pd* compared to those fed the FO and RO diet. Fish fed the FO diet had higher relative expression of *dgat-2* and *fas* than those fed the other diets, and the lowest relative expression of *dgat-2* and *fas* were observed in fish fed the SO diet.

## Discussion

The results of the present study showed that both SO and PO had the same efficiency as fish oil in improving growth performance under high dietary fishmeal supplementation. These results are consistent with previous studies in different fish species [4, 9, 41–45]. For instance, Turchini et al. [21] indicated that 100% of dietary fish oil can be substituted with VOs in fish whose EFA requirements can be satisfied by C_18_ PUFA (freshwater and salmonid species) without affecting growth performance or feed efficiency. Furthermore, most studies demonstrated that FO could be partially replaced by VOs [5, 7–8, 46–53].

FO has the highest level of LC-PUFA, such as EPA, ARA and DHA, while SO, RO and PO are all rich in linoleic acid (18:2n-6), whereas LO is rich in linolenic acid (18:3n-3). In addition, the determination of the digestibility of lipid and fatty acids from the different oil sources is important when formulating diets, with lower growth performances possibly attributed to the poor digestibility of these fatty acids [54–55]. In the present study, fish fed the diet containing LO and RO had a significantly lower WG and SGR than those fed the FO, SO and PO diets, indicating that large yellow croaker might have a low capacity to convert linoleic acid or linolenic acid into arachidonic (ARA), EPA and DHA which are essential for marine fish. The supplemental proportion of fishmeal in the diets was 41%, which would generally supply at least 4% of lipid and around 1.0–1.5% of n-3 LC-PUFA to the total diet which may be sufficient to satisfy EFA requirements. Zuo et al. [24] reported that the optimum dietary n-3 HUFA level ranged from 0.60 to 0.98% for large yellow croaker. Drew et al. [56] demonstrated that there was no effect on growth with 100% substitution of FO with VO when the dietary level of FM was over 40% in rainbow trout (*Oncorhynchus mykiss*) diets, but growth retardation was observed when fishmeal reduced to 20%. However, even total substitution (100%) of fish oil with vegetable oil (rapeseed, linseed and palm oil) in plant protein-rich diets of gilthead sea bream exhibited excellent growth [18]. The difference in the maximum level of substitution in comparison to freshwater or salmonid fish has generally been explained by the qualitative differences in EFA requirement [1]. With species that require LC-PUFA (most marine fish), the situation is slightly more complicated, and growth retardation at high supplementation of VOs requires more explanation.

It has been demonstrated that the fatty acid composition of fish is largely determined by the digestible fatty acid intake of the animal, and the relative proportions of these fatty acids are reflected in the fatty acid composition of TAGs deposited [49, 54–55]. In the present study, fish fed the FO diet had significantly higher lipid content in whole body than those fed the other diets. The lowest lipid content in whole body was observed in fish fed the diets containing LO and RO, whereas the lowest muscle lipid content occurred in fish fed the SO diet. Similar results have been found in other fish species [42–44, 57]. Some studies indicated that FO replaced by VOs also resulted in crude lipid contents of whole body and muscle increased [58–60]. However, Wang et al. [4] reported that crude lipid contents in whole body and muscle were not significantly influenced by the dietary lipid sources. Previous studies have demonstrated that SFAs are easier to deposit in tissues than monounsaturated fatty acid (MUFA) and PUFA [61]. Fish fed the LO diet had higher whole body protein levels than the other dietary treatments, while fish fed the diet containing LO had a significantly lower whole body protein content compared to those fed the FO diet [5]. However, this may attribute to different fish species, feed composition (especially fatty acid composition) and feeding strategy [62–63].

The fatty acid composition of fish muscle and liver reflected the fatty acid profile of the dietary lipids. The inclusion of VOs in fish feeds may significantly affect fish muscle quality and sensory characteristics, while some effect on the odour active compounds is also possible [19]. In the present study, fish fed the FO diet had a higher EPA and DHA contents in muscle and liver than those fed the other diets, however, fish fed the diet containing LO had higher total n-3 PUFA in muscle and liver than those fed the VOs diets. Fish fed the SO diet had a higher total n-6 PUFA in muscle and liver than those fed the other diets, whereas the lowest linoleic acid and total n-6 PUFA in liver and muscle was observed in fish fed the FO diet. Similar results have been previously observed in large yellow croaker [4, 38]. Moreover, some studies found that using 60% VO inclusion altered the nutritional quality of muscle in European sea bass (*Dicentrarchus labrax*) and gilthead sea bream (*Sparus aurata*), decreasing the level of n-3 PUFA, especially EPA and DHA as well as increasing the levels of C_18_ fatty acids such as linolenic, linoleic and oleic acids [51,64–67]. However, other studies found increased levels of linoleic acid in gilthead sea bream muscle but reduced levels of linolenic acid when fish were fed with diets containing 60% or 80% linoleic acid [51]. The replacement of marine FOs with alternative oils of vegetable or animal origin in aquafeeds needs to be studied to not only supply lipids at the correct level with the proper balance of essential fatty acids (EFAs) for optimum growth, but also to maintain the proper sensory characteristics, which contributes to the long term sustainability of the aquaculture industry [13, 68].

AST and ALT activities are often used as general indicators of the function of the vertebrate liver. In the present study, the lowest activities of AST and ALT in plasma were observed in the FO group, while fish fed the diet containing RO had higher plasma AST activity than those fed the other diets. Among all the different VO diets used, fish fed the SO diet exhibited the lowest plasma AST and ALT activities. These results suggest that FO and SO supplementation in aquafeeds could improve the normal hepatic functions of large yellow croaker. General cellular damage in mammals is usually monitored by means of analyzing leakage of cellular enzymes like AST and ALT into the blood, and similar mechanisms have also been found in other fish species [69–70]. In the current study, plasma SOD activity and MDA concentration in large yellow croaker were not significantly influenced by the different lipid sources. Similar results have been shown for other fish species [71–72]. These results indicate that, similar to FO, VOs have no negative impact to the antioxidant status.

Dietary fatty acid composition could influence lipid metabolism in fish [73]. Liver is the major tissue for lipid metabolism and plays an important metabolic role. ATGL, CPT-1 and LPL are three major enzymes involved in the lipid catabolic metabolism [74–75]. In the present study, fish fed the diets containing PO and RO had higher relative expression of *lpl* than those fed the other diets, and the lowest relative expression of *lpl* was observed in fish fed the SO diet. These results showed that the PO and RO could up-regulate the *lpl* gene expression, and that SO could down-regulate the *lpl* gene expression. Similar results have been found for hybrid tilapia [76]. The lowest ATGL activity in liver occurred in fish fed the diet containing LO, however, fish fed the LO diet also had a higher CPT-1 activity in liver than those fed the FO, SO and RO diets. Moreover, fish fed the PO diet had higher *atgl* and *cpt-1* gene expression levels than those fed the other diets. The lower ATGL activity could decrease fatty acid uptake in liver, which may imply inactive lipid metabolism. These results indicated that the lower total SFA in the LO diet could lead to a decline in ATGL activities, subsequently affecting the fatty acid composition in liver and muscle.

Triacylglycerol synthesis and catabolism are also pivotal factors affecting lipid accumulation for a specific tissue [77]. Fatty acid synthesis genes, such as *fas*, *g6pd* and *dgat-2*, are involved in lipid synthesis [28, 78]. The results of the present study showed that fish fed the FO diet up-regulated the gene expression of *dgat*-2, *fas* and *g6pd*. However, fish fed the diet containing SO down-regulated the gene expression of *dgat*-2, *fas* and *g6pd*. These results suggest that FO could promote lipid synthesis metabolism, thereby enhancing lipid deposition in tissue. This is probably the main reason that fish fed the FO diet had higher lipid content in whole body and muscle. Fish fed the VO diets displayed lower gene expression of *dgat*-2 than fish fed the FO diet. This result may reveal that reduced *dgat-2* gene expression could partially be due to a feedback mechanism involving excessive lipid accumulation in the liver, which has been observed in a mouse model of high-fat diet-induced obesity [79]. Many studies have shown that dietary fatty acid composition and lipid levels can affect lipogenesis and lipolysis [37]. The inconformity between lipid related-metabolism enzyme activities and gene expression levels should be deeply explored in future studies.

## Conclusion

The present study indicated that fish oil can be totally replaced by SO and PO in high fishmeal supplementation diets without compromising growth performance. Different lipid sources could regulate various metabolic pathways to affect the fatty acid composition in liver and muscle. In fish fed the FO diet, both increased hepatic fatty acid synthetic gene expression and decreased gene expression related to fatty acid β-oxidation may contribute to the increased lipid deposition in muscle and liver. On the contrary, in fish fed VO diets, increased gene expression related to hepatic fatty acid catabolism and β-oxidation can potentially explain the decreased lipid deposition in muscle and liver. Therefore, future studies are needed to investigate the effects of dietary lipid sources on post-translational regulation of hepatic lipid metabolism as well as the regulation mechanisms of lipogenesis and lipolysis.

## References

[1] National Research Council (2011) Committee on the Nutrient Requirements of Fish and Shrimp Nutrient requirements of fish and shrimp. Washington, DC: National Academies Press.

[2] Tocher DR (2003) Metabolism and functions of lipids and fatty acids in teleost fish. Rev Fish Sci 11: 107–184.

[3] Tacon AGJ, Metian M (2008) Global overview on the use of fish meal and fish oil in industrially compounded aquafeeds: Trends and future prospects. Aquaculture 285: 146–158.

[4] Wang XX, Li YJ, Hou CL, Gao Y, Wang YZ (2012) Influence of different dietary lipid sources on the growth, tissue fatty acid composition, histological changes and peroxisome proliferator-activated receptor γ gene expression in large yellow croaker (*Pseudosciaena crocea* R.). Aquacult Res 43: 281–291.

[5] Peng M, Xu W, Mai KS, Zhou HH, Zhang YJ, Liufu ZG, et al (2014) Growth performance, lipid deposition and hepatic lipid metabolism related gene expression in juvenile turbot (*Scophthalmus maximus* L.) fed diets with various fish oil substitution levels by soybean oil. Aquaculture 433: 442–449.

[6] Gunstone FD, Giovanni MT, Wing-Keong N, Douglas RD (2010) The world's oils and fats. Boca Raton, Florida: Taylor and Francis Group pp. 61–94.

[7] Fountoulaki E, Vasilaki A, Hurtado R, Grigorakis K, Karacostas I, Nengas I, et al (2009) Fish oil substitution by vegetable oils in commercial diets for gilthead sea bream (*Sparus aurata L*): effects on growth performance, flesh quality and fillet fatty acid profile: Recovery of fatty acid profiles by a fish oil finishing diet under fluctuating water temperatures. Aquaculture 289(3–4): 317–326.

[8] Menoyo D, Izquierdo MS, Robaina L, Ginés R, Lopez-Bote CJ, Bautista JM (2004) Adaptation of lipid metabolism, tissue composition and flesh quality in gilthead sea bream (*Sparus aurata*) to the replacement of dietary fish oil by linseed and soybean oils. Br J Nutr 92(01): 41–52. 15230986 10.1079/BJN20041165

[9] Piedecausa M, Mazón M, García B, Hernandez MD (2007) Effects of total replacement of fish oil by vegetable oils in the diets of sharp snout seabream (*Diplodus puntazzo*). Aquaculture 263: 211–219.

[10] Regost C, Arzel J, Robin J, Rosenlund G, Kaushik SJ (2003) Total replacement of fish oil by soybean or linseed oil with a return to fish oil in turbot (*Psetta maxima*): 1. Growth performance, flesh fatty acid profile, and lipid metabolism. Aquaculture 217(1): 465–482.

[11] Trushenski J, Schwarz M, Lewis H, Laporte J, Delbos B, Takeuchi R, et al (2011) Effect of replacing dietary fish oil with soybean oil on production performance and fillet lipid and fatty acid composition of juvenile cobia *Rachycentron canadum*. Aquacult Nutr 17(2): 437–447.

[12] Xu Z, Li YS, Wang J., Wu B, Li JH (2012) Effect of omega-3 polyunsaturated fatty acids to reverse biopsy-proven parenteral nutrition-associated liver disease in adults. Clinical Nutr 31(2): 217–223. 10.1016/j.clnu.2011.10.001 22035955

[13] Guillou A, Soucy P, Halil M (1995) Effects of dietary vegetable and marine lipid on growth, muscle fatty acid composition and organoleptic quality of flesh of brook charr (*Salvelinus fontinalis*). Aquaculture 136(3/4): 351–362.

[14] Tocher DR, Bell JG, Dick JR (2000) Polyunsaturated fatty acid metabolism in Atlantic salmon (*Salmo salar*) undergoing parr-smolt transformation and the effects of dietary linseed and rapeseed oils. Fish Physio Biochem 23(1): 59–73.

[15] Bell JG, Tocher DR, Henderson RJ (2003) Altered fatty acid compositions in Atlantic salmon (*Salmo salar*) fed diets containing linseed and rapeseed oils can be partially restored by a subsequent fish oil finishing diet. J Nutr 133 (9): 2793–2801. 12949367 10.1093/jn/133.9.2793

[16] Torstensen BE, Yland FR, Lie Ø (2004) Replacing dietary fish oil with increasing levels of rapeseed oil and olive oil-effects on Atlantic salmon (*Salmo salar L*.) tissue and lipoprotein lipid composition and lipogenic enzyme activities. Aquacult Nutr 10 (3):175–192.

[17] Wang JT, Han T, Tian LX (2007) Impact of three vegetable oil sources on growth, body composition and tissue fatty acid composition of juvenile cobia (*Rachycentron canadum*). J Zhejiang Ocean Univ (Natural Science) 26(3): 237–246 (In Chinese, with English abstract).

[18] Benedito-Palos L, Navarro JC, Sitjà-Bobadilla A, Bell JG, Kaushik S, Perez-Sanchez J (2008) High levels of vegetable oils in plant protein-rich diets fed to gilthead sea bream (*Sparus aurata L*): growth performance, muscle fatty acid profiles and histological alterations of target tissues. Br J Nutr 100(5): 992–1003. 10.1017/S0007114508966071 18377678

[19] Nasopoulou C, Zabetakis I (2012) Benefits of fish oil replacement by plant originated oils in compounded fish feeds. LWT-Food Sci Technol 47: 217–224.

[20] Wassef EA, Saleh NE, El-Hady HAE (2009) Vegetable oil blend as alternative lipid resources in diets for gilthead seabream, *Sparus aurata*. Aquacult Int 17(5): 421–435.

[21] Turchini GM, Torstensen BE, Ng WK (2009) Fish oil replacement in finfish nutrition. Rev Aquacult 1(1): 10–57.

[22] Ji WJ (1999) The influence of different fat sources in feed on the growth and fatty acid composition of body fat of black sea beram (*Sparus macrocephalus*). Mar Fisher Res 20(1): 70–74 (In Chinese, with English abstract).

[23] Castro C, Corraze G, Firmino-Diógenes A, Larroquet L, Panserat S, Oliva-Teles A (2016) Regulation of glucose and lipid metabolism by dietary carbohydrate levels and lipid sources in gilthead sea bream juveniles.Br J Nutr 116(1): 19–34. 10.1017/S000711451600163X 27160810

[24] Menoyo D, Izquierdo MS, Robaina L, Ginés R, Lopez-Bote CJ, Bautista JM (2004) Adaptation of lipid metabolism, tissue composition and flesh quality in gilthead sea bream (*Sparus aurata*) to the replacement of dietary fish oil by linseed and soybean oils. Br J Nutr 92: 41–52. 10.1079/BJN20041165 15230986

[25] Panserat S, Kolditz C, Richard N, Plagnes-Juan E, Piumi F, Esquerré D, et al (2008) Hepatic gene expression profiles in juvenile rainbow trout (*Oncorhynchus mykiss*) fed fishmeal or fish oil-free diets. Br J Nutr 100: 953–967. 10.1017/S0007114508981411 18439330

[26] Morais S, Pratoomyot J, Taggart JB, Plagnes-Juan E, Piumi F, Esquerré D, et al (2011) Genotype specific responses in Atlantic salmon (*Salmo salar*) subject to dietary fish oil replacement by vegetable oil: a liver transcriptomic analysis. BMC Genomics 12: 255 10.1186/1471-2164-12-255 21599965 PMC3113789

[27] Morais S, Silva T, Cordeiro O, Rodrigues P, Guy DR, Bron JE, et al (2012). Effects of genotype and dietary fish oil replacement with vegetable oil on the intestinal transcriptome and proteome of Atlantic salmon (*Salmo salar*). BMC Genomics 13: 448 10.1186/1471-2164-13-448 22943471 PMC3460786

[28] Liang X., Ogata HY, Oku H, (2002) Effect of dietary fatty acids on lipoprotein lipase gene expression in the liver and visceral adipose tissue of fed and starved red sea bream *Pagrus major*. Comp Biochem Physiol A: Mole Int Physiol 132(4): 913–919. 10.1016/s1095-6433(02)00118-6 12095871

[29] Stubhaug I, Froyland L, Torstensen BE (2005) Beta-oxidation capacity of red and white muscle and liver in Atlantic salmon (*Salmo salar* L.)–effects of increasing dietary rapeseed oil and olive oil to replace capelin oil. Lipids 40: 39–47. 15825829 10.1007/s11745-005-1358-4

[30] Morak M, Schmidinger H, Riesenhuber G, Rechberger GN, Kollroser M, Haemmerle G, et al (2012) Adipose triglyeride lipase (ATGL) and hormone-sensitive lipase (HSL) deficiencies affect expression of lipolytic activities in mouse adipose tissues. Mol Cell Proteomics 11(12): 1777–1789. 10.1074/mcp.M111.015743 22984285 PMC3518122

[31] Cases S, Smith SJ., Zheng YW, Myers HM, Lear SR, Sande E, et al (1998) Identification of a gene encoding an acyl CoA: diacylglycerol acyltransferase, a key enzyme in triacylglycerol synthesis. Proc Natl AcadSci USA 95(22): 13018–13023. 10.1073/pnas.95.22.13018 PMC23692 9789033

[32] Kroon JT, Wei W, Simon WJ, Slabas AR (2006) Identification and functional expression of a type 2 acyl-CoA: diacylglycerol acyltransferase (DGAT2) in developing castor bean seeds which has high homology to the major triglyceride biosynthetic enzyme of fungi and animals. Phytochemistry 67(23): 2541–2549. 10.1016/j.phytochem.2006.09.020 17084870

[33] Zhou PP, Wang MQ, Xie FJ, Deng DF, Zhou QC (2016) Effects of dietary carbohydrate to lipid ratios on growth performance, digestive enzyme and hepatic carbohydrate metabolic enzyme activities of large yellow croaker (*Larmichthys crocea*). Aquaculture 452: 35–51.

[34] Zuo RT, Ai QH, Mai KS, Xu W, Wang J, Xu HG, et al (2012a) Effects of dietary n-3 highly unsaturated fatty acids on growth, nonspecific immunity, expression of some immune related genes and disease resistance of large yellow croaker (*Larmichthys crocea*) following natural infestation of parasites (*Cryptocaryon irritans*). Fish Shellfish Immunol 32(2): 249–258. 22126857 10.1016/j.fsi.2011.11.005

[35] Zuo RT, Ai QH, Mai KS, Xu W, Wang J, Xu HG, et al (2012b) Effects of dietary docosahexaenoic to eicosapentaenoic acid ratio (DHA/EPA) on growth, nonspecific immunity, expression of some immune related genes and disease resistance of large yellow croaker (*Larmichthys crocea*) following natural infestation of parasites (*Cryptocaryon irritans*). Aquaculture 334-337(1): 101–109. 10.1016/j.fsi.2011.11.005 22126857

[36] Yi XW, Zhang F, Xu W, Li J, Zhang WB, Mai KS (2014) Effects of dietary lipid content on growth, body composition and pigmentation of large yellow croaker *Larimichthys croceus*. Aquaculture 434: 355–361.

[37] Yan J, Liao K, Wang TJ, Mai KS, Xu W, Ai QH (2015) Dietary lipid levels influence lipid deposition in the liver of large yellow croaker (*Larimichthys crocea*) by regulating lipoprotein receptors, fatty acid uptake and triacylglycerol synthesis and catabolism at the transcriptional level. Plos one 10(6) e0129937 10.1371/journal.pone.0129937 26114429 PMC4482732

[38] Yi XW, Zhang WB, Mai KS, Shen-Tu JK (2013) Effects of dietary fish oil replaced with rapeseed oil on the growth, fatty acid composition and skin color of large yellow croaker (*Larimichthys crocea*). J Fish Chin 37 (5): 751–761 (In Chinese, with English abstract).

[39] Association of Official Analytical Chemists (1995) Official Methods of Analysis of Official Analytical Chemists International, 16th ed Association of Official Analytical Chemists, Arlington, VA.

[40] Livak KJ, Schmittgen TD (2001) Analysis of Relative Gene Expression Data Using Real-Time QuantitativePCR and the 2^−ΔΔCT^ Method. Methods 25: 402–408. 10.1006/meth.2001.1262 11846609

[41] Richard N, Mourente G, Kaushik S, Corraze G (2006) Replacement of a large portion of fish oil by vegetable oils does not affect lipogenesis, lipid transport and tissue lipid uptake in European seabass (*Dicentrarchus labrax* L.). Aquaculture 261: 1077–1087.

[42] Bell JG, McEvoy J, Tocher DR, McGhee F, Campbell PJ, Sargent JR (2001) Replacement of fish oil with rapeseed oil in diets of Atlantic salmon (*Salmo salar*) affects tissue lipid compositions and hepatocyte fatty acid metabolism. Br J Nutr 131: 1535–1543. 10.1093/jn/131.5.1535 11340112

[43] Bell JG, Henderson RJ, Tocher DR, McGhee F, Dick JR, Porter A, et al (2002) Substituting fish oil with crude palm oil in the diet of Atlantic salmon (*Salmo salar*) affects muscle fatty acid composition and hepatic fatty acid metabolism. Br J Nutr 132: 222–230. 10.1093/jn/132.2.222 11823582

[44] Nanton DA, Vegusdal A, Rora AMB, Ruyter B, Baeverfjord G, Torstensen BE (2007) Muscle lipid storage pattern, composition, and adipocyte distribution in different parts of Atlantic salmon (*Salmo salar*) fed fish oil and vegetable oil. Aquaculture 265: 230–243.

[45] Rosenlund G, Obach A, Sandberg M, Standal H, Tveit K (2001) Effect of alternative lipid sources on long-term growth performance and quality of Atlantic salmon (*Salmo salar* L.). Aquacult Res 32: 323–328.

[46] Bell JG, McGhee F, Dick JR, Tocher DR (2005) Dioxin and dioxin-like polychlorinated biphenyls (PCBs) in Scottish farmed salmon (*Salmo salar*): effects of replacement of dietary marine fish oil with vegetable oils. Aquaculture 243: 305–314.

[47] Caballero M J, Obach A, Rosenlund G (2002) Impact of different dietary lipid sources on growth, lipid digestibility, tissue fatty acid composition and histology of rainbow trout, *Oncorhynchus mykiss*. Aquaculture 214(1–4): 253–271.

[48] Figueiredo-Silva A, Rocha E, Dias J, Silva P, Rema P, Gomes E, et al (2005) Partial replacement of fish oil by soybean oil on lipid distribution and liver histology in European sea bass (*Dicentrarchus labrax*) and rainbow trout (*Oncorhynchus mykiss*) juveniles. Aquacult Nutr 11(2): 147–155.

[49] Greene DH, Selivonchick DP (1990) Effects of dietary vegetable, animal and marine lipids on muscle lipid, and hematology of rainbow trout (*Oncorhynchus mykiss*). Aquaculture 89: 165–182.

[50] Hardy RW, Scott TM, Harrell LW (1987) Replacement of herring oil with menhaden oil, soybean oil, or tallow in the diets of Atlantic salmon raised in marine net-pens. Aquaculture 65: 267–277.

[51] Izquierdo MS, Montero D, Robaina L, Caballero MJ, Rosenlund G, Gines R (2005) Alterations in fillet fatty acid profile and flesh quality in gilthead seabream (*Sparus aurata*) fed vegetable oils for a long term period. Recovery of fatty acid profiles by fish oil feeding. Aquaculture 250(1/2): 431–444.

[52] Trushenski J, Schwarz M, Lewis H, Laporte J, Delbos B, Takeuchi R, et al (2011) Effect of replacing dietary fish oil with soybean oil on production performance and fillet lipid and fatty acid composition of juvenile cobia *Rachycentron canadum*. Aquacult Nutr 17(2): 437–447.

[53] Xu Z, Li YS, Wang, Wu B, Li JH (2012) Effect of omega-3 polyunsaturated fatty acids to reverse biopsy-proven parenteral nutrition-associated liver disease in adults. Clinical Nutr 31(2): 217–223. 10.1016/j.clnu.2011.10.001 22035955

[54] Sarker PK, Kapuscinski AR, Lanois AJ, Livesey ED, Bernhard K, Coley ML (2016) Towards sustainable aquafeeds: complete substitution of fish oil with marine microalga *schizochytrium* sp. Improves growth and fatty acid deposition in juvenile Nile tilapia (*Oreochromisniloticus*). PloS one 11(6) e0156684 10.1371/journal.pone.0156684 27258552 PMC4892564

[55] Sarker PK, Gamble MM, Kelson S, Kapuscinski ARK (2016) Nile tilapia (*Oreochromis niloticus*) show high digestibility of lipid and fatty acids from marine *Schizochytrium* sp. and of protein and essential amino acids from freshwater *Spirulina* sp. feed ingredients. Aquacult Nutr 22: 109–119.

[56] Drew MD, Ogunkoya AE, Janz DM, Van Kessel AG (2007) Dietary influence of replacing fish meal and oil with canola protein concentrate and vegetable oils on growth performance, fatty acid composition and organochlorine residues in rainbow trout (*Oncorhynchus mykiss*). Aquaculture 267(1): 260–268.

[57] Subhadra B, Lochmann R, Rawles S, Chen RG (2006) Effect of dietary lipid source on the growth, tissue composition and hematological parameters of largemouth bass (*Micropterus salmoides*). Aquaculture 263: 211–219.

[58] Cruz-Garcia L, Sanchez-Gurmaches J, Bouraoui L (2011) Effects of dietary fish meal and fish oil replacement on lipogenic and lipoprotion lipase activities and plasma insulin in gilthead sea beram (*Sparus aurata*). Aquaculture 17: 54–63.

[59] Menoyo D, Diez A, Lopez-Bote CJ, Casado S, Obach A, Bautista JM (2006) Dietary fat type affects lipid metabolism in Atlantic salmon (*Salmo salar* L.) and differentially regulates glucose transporter GLUT4 expression in muscle. Aquaculture 261: 294–304.

[60] Torstensen BE, Espe M, Stubhaug I (2011) Dietary plant proteins and vegetable oil blends increase adiposity and plasma lipids in Atlantic salmon (*Salmo salar L*.). Br J Nutr 106: 633–647. 10.1017/S0007114511000729 21535902

[61] Clarke SD, Armstrong MK, Jump DB (1990) Dietary polyunsaturated fats uniquely suppress rat liver fatty acid synthase and s14 mRNA content. Br J Nutr 120: 225–231. 10.1093/jn/120.2.225 2313387

[62] Borges P, Oliveira B, Casal S, Dias J, Conceiçao L, Valente LM (2009) Dietary lipid level affects growth performance and nutrient utilization of Senegalese sole (*Solea senegalensis*) juveniles. Br J Nutr 102: 1007–1014. 10.1017/S0007114509345262 19393115

[63] Ng WK, Campbell PJ, Dick JR, Bell JG (2003) Interactive effects of dietary palm oil concentration and water temperature on lipid digestibility in rainbow trout, *Oncorhynchus mykiss*. Lipids 38: 1031–1038. 14669967 10.1007/s11745-006-1157-y

[64] Izquierdo MS, Obach A, Arantzamendi L, Montero D, Robaina L, Rosenlund G (2003) Dietary lipid sources for seabream and seabass: growth performance, tissue composition and flesh quality. Aquacult Nutr 9(6): 397–407.

[65] Mourente G, Bell JG (2006) Partial replacement of dietary fish oil with blends of vegetable oils (rapeseed, linseed and palm oils) in diets for European sea bass (*Dicentrarchus labrax L*.) over a long term growth study: effects on muscle and liver fatty acid composition and effectiveness of a fish oil finishing diet. Comp. Biochem Physiol B: Biochem Mol Biol 145(3–4): 389–399. 17055762 10.1016/j.cbpb.2006.08.012

[66] Montero D, Robaina L, Caballero MJ, Ginés R, Izquierdo MS (2005) Growth, feed utilization and flesh quality of European sea bass (*Dicentrarchus labrax*) fed diets containing vegetable oils: a time-course study on the effect of a re-feeding period with a 100% fish oil diet. Aquaculture 248(1): 121–134.

[67] Mourente G, Dick JR, Bell JG, Tocher DR (2005) Effect of partial substitution of dietary fish oil by vegetable oils on desaturation and b-oxidation of [1-14C] 18:3n-3 (LNA) and [1-14C] 20:5n-3 (EPA) in hepatocytes and enterocytes of European sea bass (*Dicentrarchus labrax* L.). Aquaculture 248(1–4): 173–186.

[68] Hardy RW (2010) Utilization of plant proteins in fish diets: effects of global demand and supplies of fishmeal. Aquacult Res 41(5): 770–776.

[69] Welker TL, Congleton JL (2003) Relationship between dietary lipid source, oxidative stress, and the physiological response to stress in sub-yearling chinook salmon (*Oncorhynchus tshawytscha*). Fish Physiol Biochem 29: 225–235.

[70] Olsen RE, Sundell K, Mayhew TM, Myklebust R, Ringø E (2005) Acute stress alters intestinal function of rainbow trout, *Oncorhynchus mykiss* (W). Aquaculture 250: 480–495.

[71] Calvin MN, Muscatine L (1997) Oxidative stress in the symbiotic sea anemone *Aiptasia pulchella* (Carlgren, 1943): contribution of the animal to superoxide ion production at elevated temperature. Biol Bulletin 192: 444–456. 10.2307/1542753 28581838

[72] Lin SM, Pan Y, Luo L, Luo L (2011) Effects of dietary β-1,3-glucan, chitosan or raffinose on the growth, innate immunity and resistance of koi (*Cyprinus carpio* koi). Fish Shellfish Immunol 31: 788–794. 10.1016/j.fsi.2011.07.013 21784160

[73] Gonzalez-Duran E, Castell JD, Robinson SMC, Blair TJ (2008) Effects of dietary lipids on the fatty acid composition and lipid metabolism of the green sea urchin *Strongylocentrotus droebachiensis*. Aquaculture 276(1–4): 120–129.

[74] Kerner J, Hoppel C (2000) Fatty acid import into mitochondria. Biochim Biophy Acta (BBA)–Molec. Cell Biol Lipids 1486: 1–17. 10.1016/s1388-1981(00)00044-5 10856709

[75] Cowey CB, Walton MJ (1989) Intermediary metabolism. Fish Nutr 2: 259–329.

[76] Han CY, Zheng QM, Feng LN (2013) Effects of total replacement of dietary fish oil on growth performance and fatty acid compositions of hybrid tilapia (*Oreochromis niloticus×O*. *areus*). Aquacult Int 21: 1209–1217.

[77] Li XF, Jiang GZ, Qian Y, Xu WN, Liu WB (2013) Molecular characterization of lipoprotein lipase from blunt snout bream *Megalobrama amblycephala* and the regulation of its activity and expression by dietary lipid levels. Aquaculture 416: 23–32.

[78] Yen CLE, Stone SJ, Koliwad S, Harris C, Farese RV (2008) Thematic review series: glycerolipids. DGAT enzymes and triacylglycerol biosynthesis. Lipid Res 49:2283–2301. 10.1194/jlr.R800018-JLR200 PMC3837458 18757836

[79] Kim S, Sohn I, Ahn JI, Lee KH, Lee YS (2004) Hepatic gene expression profiles in a long term high-fat diet-induced obesity mouse model. Gene 340: 99–109. 10.1016/j.gene.2004.06.015 15556298

